# Immune correlates analysis of a phase 3 trial of the AZD1222 (ChAdOx1 nCoV-19) vaccine

**DOI:** 10.1038/s41541-023-00630-0

**Published:** 2023-03-11

**Authors:** David Benkeser, Youyi Fong, Holly E. Janes, Elizabeth J. Kelly, Ian Hirsch, Stephanie Sproule, Ann Marie Stanley, Jill Maaske, Tonya Villafana, Christopher R. Houchens, Karen Martins, Lakshmi Jayashankar, Flora Castellino, Victor Ayala, Christos J. Petropoulos, Andrew Leith, Deanne Haugaard, Bill Webb, Yiwen Lu, Chenchen Yu, Bhavesh Borate, Lars W. P. van der Laan, Nima S. Hejazi, Lindsay N. Carpp, April K. Randhawa, Michele P. Andrasik, James G. Kublin, Margaret Brewinski Isaacs, Mamodikoe Makhene, Tina Tong, Merlin L. Robb, Lawrence Corey, Kathleen M. Neuzil, Dean Follmann, Corey Hoffman, Ann R. Falsey, Magdalena Sobieszczyk, Richard A. Koup, Ruben O. Donis, Peter B. Gilbert, on behalf of the AstraZeneca AZD1222 Clinical Study Group, the Immune Assays Team, the United States Government (USG)/CoVPN Biostatistics Team

**Affiliations:** 1 Department of Biostatistics and Bioinformatics, Rollins School of Public Health, Emory University, Atlanta, GA USA; 2 Vaccine and Infectious Disease Division, Fred Hutchinson Cancer Center, Seattle, WA USA; 3 Public Health Sciences Division, Fred Hutchinson Cancer Center, Seattle, WA USA; 4 Translational Medicine, Vaccines & Immune Therapies, BioPharmaceuticals R&D, AstraZeneca, Gaithersburg, MD USA; 5 Biometrics, Vaccines & Immune Therapies, BioPharmaceuticals R&D, AstraZeneca, Cambridge, United Kingdom; 6 Biometrics, Vaccines & Immune Therapies, BioPharmaceuticals R&D, AstraZeneca, Gaithersburg, MD USA; 7 Clinical Development, Vaccines & Immune Therapies, BioPharmaceuticals R&D, AstraZeneca, Gaithersburg, MD USA; 8 Biomedical Advanced Research and Development Authority, Administration for Strategic Preparedness and Response, Department of Health and Human Services, Washington, DC USA; 9 LabCorp-Monogram Biosciences, South San Francisco, CA USA; 10 Nexelis, Seattle, WA USA; 11 Department of Statistics, University of Washington, Seattle, WA USA; 12 Department of Biostatistics, T.H. Chan School of Public Health, Harvard University, Boston, MA USA; 13 Division of AIDS, National Institute of Allergy and Infectious Diseases, Bethesda, MD USA; 14 Division of Microbiology and Infectious Diseases, National Institute of Allergy and Infectious Diseases, National Institutes of Health, Bethesda, MD USA; 15 Vaccine Translational Research Branch, National Institute of Allergy and Infectious Diseases, National Institutes of Health, Rockville, MD USA; 16 US Military HIV Research Program, Walter Reed Army Institute of Research, Silver Spring, MD USA; 17 Department of Laboratory Medicine and Pathology, University of Washington, Seattle, WA USA; 18 Center for Vaccine Development and Global Health, University of Maryland School of Medicine, Baltimore, MD USA; 19 Biostatistics Research Branch, National Institute of Allergy and Infectious Diseases, National Institutes of Health, Bethesda, MD USA; 20 Division of Infectious Diseases, Department of Medicine, University of Rochester, Rochester, NY USA; 21 Division of Infectious Diseases, Department of Medicine, Columbia University Irving Medical Center and New York-Presbyterian Hospital, New York, NY USA; 22 Vaccine Research Center, National Institute of Allergy and Infectious Diseases, National Institutes of Health, Bethesda, MD USA; 23 Department of Biostatistics, School of Public Health, University of Washington, Seattle, WA USA

**Keywords:** Vaccines, Viral infection, Infectious diseases, Adaptive immunity

## Abstract

In the phase 3 trial of the AZD1222 (ChAdOx1 nCoV-19) vaccine conducted in the U.S., Chile, and Peru, anti-spike binding IgG concentration (spike IgG) and pseudovirus 50% neutralizing antibody titer (nAb ID50) measured four weeks after two doses were assessed as correlates of risk and protection against PCR-confirmed symptomatic SARS-CoV-2 infection (COVID-19). These analyses of SARS-CoV-2 negative participants were based on case-cohort sampling of vaccine recipients (33 COVID-19 cases by 4 months post dose two, 463 non-cases). The adjusted hazard ratio of COVID-19 was 0.32 (95% CI: 0.14, 0.76) per 10-fold increase in spike IgG concentration and 0.28 (0.10, 0.77) per 10-fold increase in nAb ID50 titer. At nAb ID50 below the limit of detection (< 2.612 IU50/ml), 10, 100, and 270 IU50/ml, vaccine efficacy was −5.8% (−651%, 75.6%), 64.9% (56.4%, 86.9%), 90.0% (55.8%, 97.6%) and 94.2% (69.4%, 99.1%). These findings provide further evidence towards defining an immune marker correlate of protection to help guide regulatory/approval decisions for COVID-19 vaccines.

## Introduction

The AZD1222 (ChAdOx1 nCoV-19) vaccine is a replication-deficient simian adenoviral vector expressing full-length wild-type SARS-CoV-2 spike protein. AZD1222 was shown to be safe and immunogenic in adults^1^ and prevented virologically confirmed symptomatic COVID-19 disease in a phase 2/3 study conducted in the United Kingdom and a phase 3 study conducted in Brazil^2–4^. Another phase 3 trial (which we refer to as the US/LatAm AZD1222 trial) conducted in the U.S., Chile, and Peru showed that two doses of AZD1222 were safe and prevented SARS-CoV-2 infection and COVID-19; the present work focuses on this trial^5^.

The US/LatAm AZD1222 trial randomized 32,451 participants in a 2:1 ratio to receive 2 doses of AZD1222 or placebo between August 28, 2020 and January 15, 2021. Based on occurrence of 203 COVID-19 primary endpoints (73 among vaccine recipients and 130 among placebo recipients at least 15 days after the second vaccine dose) over ~2 months of follow-up post second vaccination, vaccine efficacy was 74.0%; 95% confidence interval [CI], 65.3 to 80.5^5^. Whole-genome sequencing of samples from 359 participants attending illness visits showed only small numbers of variants of concern or of interest, with the predominant variant being B.1.2^5^. The AZD1222 vaccine has been issued an Emergency Use Listing by the World Health Organization^6^, conditionally authorized for use in the European Union by the European Commission^7^, and granted approval or authorization in nearly 150 countries^8^.

An immune biomarker that can be used to reliably predict vaccine efficacy against a clinical outcome is a “correlate of protection” (CoP)^9–11^. A validated CoP is highly sought in vaccine research, because it can aid and expedite decisions pertaining to approval and use. Examples of potential uses for a validated CoP include serving as a basis for approving the vaccine for populations not included in the original phase 3 trial (such as children) or for approving alternative formulations or schedules (e.g. variant-adapted versions, or alternative dosing). A validated CoP can also guide and accelerate vaccine research by providing an immunogenicity study endpoint for ranking and down-selection of candidate vaccine regimens and as a key endpoint for provisional or traditional approval of vaccines.

A growing body of evidence supports binding antibodies (bAbs) and neutralizing antibodies (nAbs) (common CoPs for many licensed vaccines^10^) as CoPs for COVID-19 vaccines^12–20^. A major objective of the five harmonized phase 3 COVID-19 vaccine efficacy trials designed and implemented by the US Government (USG) COVID-19 Response Team and the vaccine developers is to develop a CoP based on an IgG bAb or nAb assay^21^. Assay measurements included in the correlates analyses in this program are IgG bAbs against SARS-CoV-2 spike protein (“spike IgG”), IgG bAbs against the spike protein receptor binding domain (“RBD IgG”), and neutralizing antibodies measured by a pseudovirus neutralization assay (50% inhibitory dilution titer, “nAb ID50”) as CoPs. Results are reported in World Health Organization (WHO) International Units and a harmonized immune correlates Statistical Analysis Plan (SAP)^22^ is implemented to enable cross-study comparisons. Within this program, immune correlates analyses of the COVE trial of the mRNA-1273 vaccine^23^, the ENSEMBLE trial of the Ad26.COV2.S vaccine^24^, and the PREVENT-19 trial of the NVX-CoV2373 vaccine^25^ have evaluated these markers at various time points as correlates of risk of symptomatic COVID-19 in vaccine recipients and as correlates of vaccine protection^23–25^. Outside the USG-supported program, an immune correlates analysis of the COV002 trial of the AZD1222 (ChAdOx1 nCoV-19) vaccine in the United Kingdom similarly evaluated these markers^26^. Here, we assess the spike IgG and nAb ID50 markers as correlates in the US/LatAm AZD1222 trial, using the same harmonized sampling design and statistical methods, and compare our findings to those of the other USG-supported trials.

## Results

### Immunogenicity subcohort and case-cohort set

The assessment of immune correlates was based on measurement of spike IgG and nAb ID50 at D57 in the case-cohort set, comprised of a stratified random sample of the study cohort (the “immunogenicity subcohort”) plus all vaccine recipients experiencing virologically confirmed symptomatic COVID-19 seven or more days after D57 (“breakthrough cases”). All analyses of D57 antibody markers were restricted to baseline SARS-CoV-2 negative participants in the Day 57 marker case-cohort set (defined in Supplementary Table 1) (Supplementary Figs. 1 and 2) who received both planned vaccinations without any specified protocol deviations, and who were SARS-CoV-2 negative at the terminal vaccination visit. The same two markers were also assessed at D29 as immune correlates in the Day 29 marker case-cohort set, defined in parallel fashion to the Day 57 marker case-cohort set (Supplementary Table 1, Supplementary Figs. 1 and 2). D57 spike IgG antibody data were available from 33 of 45 vaccine recipient breakthrough cases and 463 vaccine recipient non-cases. D57 nAb ID50 data were available from 22 of these 33 vaccine recipient breakthrough cases and from 421 of these 463 non-cases. All results focus on the D57 markers, except the last section of Results summarizes results for the D29 markers.

### Participant demographics

Demographics and clinical characteristics of participants selected for the immunogenicity subcohort are shown in Supplementary Table 2. Of all immunogenicity subcohort participants, 47.9% were ≥65 years old, 66.6% had at least one co-existing condition (full list in Falsey et al.^5^), 40.7% were female, and 63.8%, 19.3%, and 17.0% were enrolled in the U.S., Chile, and Peru, respectively. The sampling design for the immunogenicity subcohort over-sampled participants age ≥65, with at least one co-existing condition, and minorities in the U.S. (defined as other than White Non-Hispanic). This over-sampling was accounted for in the correlates statistical analyses so that all inferences apply to nearly the same population as in Falsey et al.

### COVID-19 study endpoint

Correlates analyses were performed based on adjudicated SARS-CoV-2 RT-PCR–positive symptomatic illness endpoints, with “SARS-CoV-2 RT-PCR-positive symptomatic illness” (hereafter, COVID-19) defined as in ref. ^5^. Cases were defined as baseline SARS-CoV-2 negative participants who received both planned vaccinations without any specified protocol deviations and in whom the COVID-19 endpoint started at least 7 days post-D57 visit (for analysis of D57 markers as correlates) or at least 7 days post-D29 visit (for analysis of D29 markers as correlates), differing from Falsey et al.^5^ where onset of the COVID-19 endpoint was required to be at least 15 days post-D29 (second dose) (see the Supplementary Text for the overlap of endpoints in Falsey et al. and this correlates study). Supplementary Fig. 3 shows the timing of injections, serum sampling, and COVID-19 endpoint diagnosis. Out of the 69 and 33 COVID-19 endpoints that were included in the D29 and D57 correlates analyses, respectively, all met the Centers for Disease Control and Prevention (CDC) criteria for SARS-CoV-2 RT-PCR-positive symptomatic illness (ref. ^27^. as cited in Falsey et al.), and 68 and 32 met the University of Oxford criteria for SARS-CoV-2 RT-PCR-positive symptomatic illness (see the Supplementary Text for the definition). The correlates analyses excluded COVID-19 endpoints between 1 and 6 days post-D57 (or post-D29) visit because some of these participants likely had SARS-CoV-2 infection before the D57 (or D29) study visit, which may affect antibody levels at the study visit. In both the correlates analyses and Falsey et al., COVID-19 endpoints were included through to March 5, 2021, the data cut date of the primary analysis. Vaccine recipient non-cases for analysis of D57 (D29) correlates were defined as baseline SARS-CoV-2 negative participants who received both planned vaccinations without any specified protocol deviations and who were sampled into the immunogenicity subcohort with D57 (D29) antibody data measured with no evidence of SARS-CoV-2 infection (i.e., never tested RT-PCR positive) up to the end of the correlates study period (March 5, 2021). The cumulative probability of COVID-19 was estimated through 92 days post-dose two; this time point was selected as the latest COVID-19 event time after D57 among the vaccine breakthrough COVID-19 endpoint cases with antibody data.

### Vaccine recipient non-cases had higher D57 spike IgG concentrations and neutralization ID50 titers than vaccine breakthrough cases

**Fig. 1:**
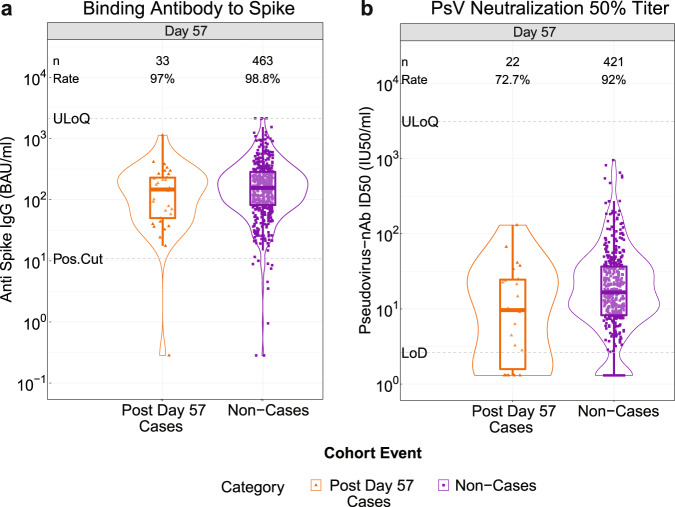
D57 antibody marker level by COVID-19 outcome status in baseline SARS-CoV-2 negative vaccine recipients. **a** Anti-spike IgG concentration and **b** pseudovirus (PsV) neutralization ID50 titer. The violin plots contain interior box plots with upper and lower horizontal edges the 25th and 75th percentiles of antibody level and middle line the 50th percentile, and vertical bars the distance from the 25th (or 75th) percentile of antibody level and the minimum (or maximum) antibody level within the 25th (or 75th) percentile of antibody level minus (or plus) 1.5 times the interquartile range. At both sides of the box, a rotated probability density curve estimated by a kernel density estimator with a default Gaussian kernel is plotted. Frequencies of participants with positive spike IgG/detectable nAb ID50 responses were computed with inverse probability of sampling weighting (reported at the top of the plots as “Rate”). Pos.Cut, Positivity cut-off for spike IgG defined by IgG >10.8424 BAU/ml, the assay positivity cut-off. ULoQ = 6934 BAU/ml for spike IgG. Seroresponse for ID50 was defined by a detectable value >limit of detection (LOD) (2.612 IU50/ml). ULoQ = 8319.938 IU50/ml. Post Day 57 cases experienced the primary COVID-19 endpoint starting 7 days post D57 visit through to the data cut (March 5, 2021). Non-cases are sampled into the immunogenicity subcohort with no evidence of SARS-CoV-2 infection (i.e., never tested RT-PCR positive) up to the end of the correlates study period (the data cut-off date March 5, 2021).

At D57, 98.8% (95% CI: 96.7%, 99.6%) of vaccine recipient non-cases had a positive spike IgG response and 92.0% (87.8%, 94.9%) had a detectable nAb ID50 titer (Fig. 1, Table 1). For both D57 markers the proportion of vaccine recipients with positive/detectable response was lower in cases than in non-cases and the geometric mean value was higher for non-cases than for cases (Table 1).

**Table 1:** D57 antibody marker^a^ SARS-CoV-2 seroresponse rates and geometric means by COVID-19 outcome status.

	Post Day 57 COVID-19 Cases^b^			Non-Cases in Immunogenicity Subcohort^c^			Comparison	
D57 Marker	N	Proportion with Antibody Response^d^ (95% CI)	Geometric Mean (GM) (95% CI)	N	Proportion with Antibody Response^d^ (95% CI)	Geometric Mean (GM) (95% CI)	Response Rate Difference (Non-Cases – Cases)	Ratio of GM (Non-Cases/ Cases)
Anti Spike IgG (BAU/ml)	33	97.0% (80.0%, 99.6%)	100.3 (62.1, 162.1)	463	98.8% (96.7%, 99.6%)	156.4 (139.2, 175.9)	1.9% (−1.5, 18.8%)	1.6 (0.95, 2.6)
Pseudovirus-nAb ID50 (IU50/ml)	22	72.7% (49.3%, 88.0%)	8.4 (4.6, 15.5)	421	92.0% (87.8%, 94.9%)	18.4 (15.5, 21.8)	19.3% (3.5, 42.9%)	2.2 (1.2, 4.2)

^a^Median (interquartile range) days from vaccination to D57 was 57 (2).

^b^Post Day 57 cases are baseline SARS-CoV-2 negative vaccine recipients who received both planned vaccinations without any specified protocol deviations and were at risk at D57 and experienced symptomatic RT-PCR-confirmed COVID-19 starting 7 days post D57 visit through to the data cut (March 5, 2021) and were hence included in the D57 correlates analyses. “*N*” refers to the number of these cases (see Supplementary Fig. 3).

^c^Non-cases are baseline negative vaccine recipients sampled into the immunogenicity subcohort with no evidence of SARS-CoV-2 infection (i.e., never tested RT-PCR positive) up to the end of the correlates study period (the data cut-off date March 5, 2021) and with D57 antibody data and hence were included in the D57 correlates analyses.

^d^Antibody response defined by IgG concentration above the assay positivity cut-off (10.8424 BAU/ml) or by detectable ID50 > limit of detection (LOD) = 2.612 IU50/ml.

Analysis based on baseline SARS-CoV-2 negative vaccine recipients in the Day 57 case-cohort set.

**Fig. 2:**
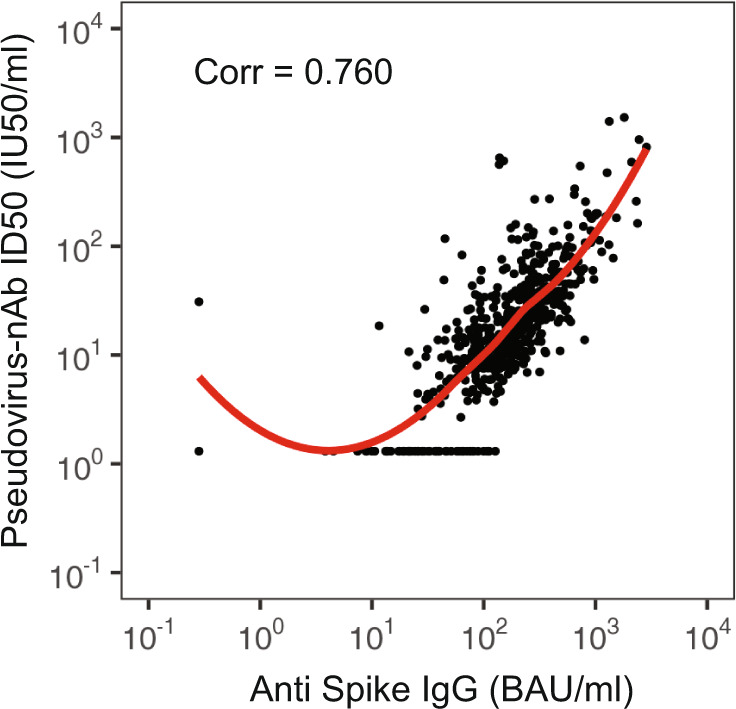
Scatterplot of D57 spike IgG vs. D57 pseudovirus (PsV)-nAb ID50 values for baseline SARS-CoV-2 negative vaccine recipients in the immunogenicity subcohort. Corr, Spearman rank correlation.

The two D57 markers were moderately-to-highly correlated (Spearman rank *r* = 0.76, Fig. 2; restricting to individuals above each assay’s positivity threshold Spearman rank *r* = 0.71). The correlations between the D29 and D57 measurements for each marker were lower (*r* = 0.54 for spike IgG, *r* = 0.45 for ID50) (Supplementary Fig. 4). For each D57 marker, the reverse cumulative distribution function curve in the context of the overall vaccine efficacy estimate is shown in Supplementary Fig. 5. As expected, because the analyzed cohort is baseline SARS-CoV-2 negative, proportions of placebo recipients with positive or detectable responses at D57 were low or zero (Supplementary Table 3).

### D57 spike IgG concentration and neutralization ID50 titer are inversely correlated with risk of COVID-19 in vaccine recipients

**Fig. 3:**
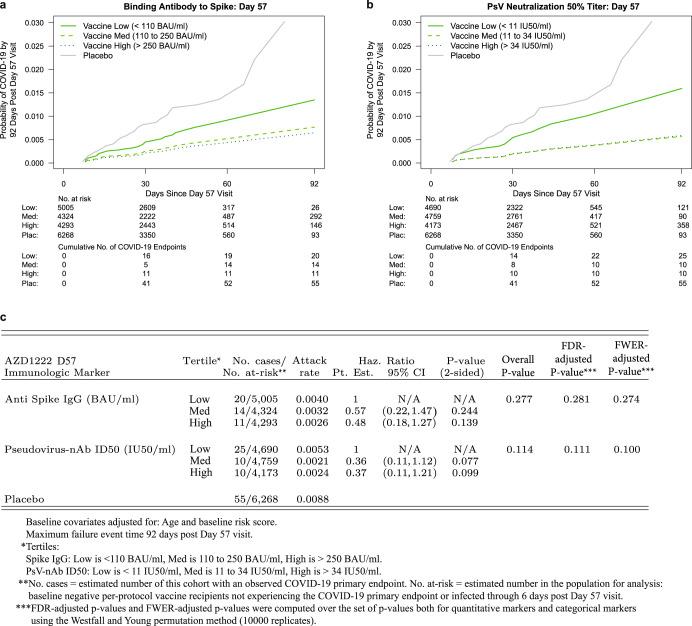
COVID-19 risk by D57 antibody marker level in baseline SARS-CoV-2 negative vaccine recipients. Cumulative incidence of COVID-19 by Low, Medium, High tertile of D57 antibody marker level. **a**, **c** Anti-spike IgG concentration; **b**, **c** pseudovirus (PsV) neutralization ID50 titer. The overall *P*-value is from a generalized Wald-test *p*-value of the null hypothesis that the hazard rate is constant across the Low, Medium, and High tertile groups. The total number of cases across the Low, Medium, and High tertiles (*N* = 45) for each antibody marker differs from the numbers in Table 1 and Fig. 1 (*N* = 33, 22 for spike IgG, PsV-nAb ID50, respectively) because the 45 includes all vaccine breakthrough cases including those without D57 antibody marker data.

The cumulative incidence of COVID-19 for vaccine recipient subgroups defined by D57 antibody marker tertiles suggest a trend for decreasing COVID-19 risk with increasing tertile, for both antibody markers (Fig. 3). There were 20, 14, and 11 breakthrough cases in the Low, Medium, and High D57 spike IgG antibody subgroups, with point estimates of marginalized hazard ratios (HRs) indicating a dose-response trend with hazard rate decreasing with increasing antibody level categories (overall *p*-value of 0.28 for the test of the null hypothesis that the hazard rate is constant across tertiles). There were 25, 10, and 10 breakthrough cases in the Low, Medium, and High D57 nAb ID50 antibody groups, with marginalized hazard ratios supporting lower risk in Medium and High compared to Low (overall *p*-value 0.11).

For the D57 markers studied as quantitative markers, both markers significantly inversely correlated with risk, with about 3-fold lower hazard rate per 10-fold increase in each antibody marker (Table 2A). Both markers exhibited strong evidence as correlates, passing multiple hypothesis testing correction. When the results were placed on the scale hazard ratio per standard deviation increase in marker value, the correlate strengths were similar (Table 2B).

**Table 2:** Hazard ratios of COVID-19 (A) per 10-fold increase or (B) per standard deviation increase in each D57 marker in baseline negative vaccine recipients.

A					
D57 Antibody Marker	Hazard Ratio per 10-fold Increase		*P*-value* (2-sided)	*Q*-value**	FWER**
	Point Est.	95% CI			
Anti Spike IgG (BAU/ml)	0.32	(0.14, 0.76)	0.009	0.017	0.015
PsV-nAb ID50 (IU50/ml)	0.28	(0.10, 0.77)	0.013	0.019	0.016

B					
D57 Antibody Marker	Hazard Ratio per Standard Deviation-Increment Increase				
	Point Est.	95% CI			
Anti Spike IgG (BAU/ml)	0.57	(0.37, 0.87)			
PsV-nAb ID50 (IU50/ml)	0.48	(0.27, 0.86)			

Analyses were adjusted for age and baseline risk score. Maximum failure event 92 days post Day 57 visit.

*P-values are not shown for B because they are structurally identical to those for A.

**Q-value and FWER (family-wise error rate) are computed over the two *p*-values for the two quantitative markers using the Westfall and Young permutation method (10,000 replicates).

**Fig. 4:**
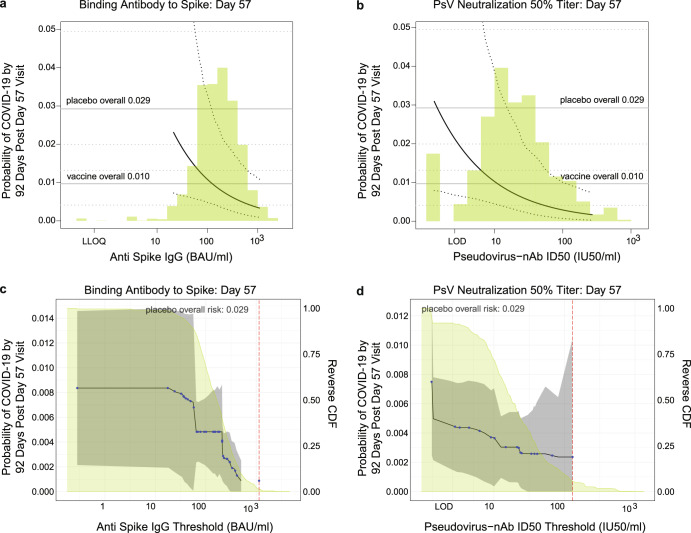
Analyses of D57 antibody markers as a correlate of risk in baseline SARS-CoV-2 negative vaccine recipients. **a**, **b** Age and baseline risk score-adjusted cumulative incidence of COVID-19 by 92 days post D57 by D57 (A) anti-spike IgG or (**b**) pseudovirus (PsV)-nAb ID50 titer, estimated using a marginalized Cox model. The dotted black lines indicate bootstrap pointwise 95% CIs. The upper and lower horizontal gray lines are the overall cumulative incidence of COVID-19 from 7 to 92 days post D57 in placebo and vaccine recipients, respectively. Curves are plotted over the antibody marker range from the 2.5th percentile to the 97.5th percentile: 21.3 to 1088 BAU/ml for spike IgG and 1.31 to 270 IU50/ml for PsV-nAb ID50. **c**, **d** Age and baseline risk score-adjusted cumulative incidence of COVID-19 by 92 days post D57 by D57 (**c**) anti-spike IgG or (**d**) PsV-nAb ID50 titer above a threshold. The blue dots are point estimates at each COVID-19 primary endpoint linearly interpolated by solid black lines; the gray shaded area is pointwise 95% confidence intervals (CIs). The estimates and CIs assume a non-increasing threshold-response function. The upper boundary of the green shaded area is the estimate of the reverse cumulative distribution function (CDF) of D57 antibody marker level. The vertical red dashed line is the D57 antibody marker threshold above which no COVID-19 endpoints occurred (in the time frame of 7 days post D57 through to the data cut-off date March 5, 2021). PsV, pseudovirus.

Panels A and B in Fig. 4 show the marginalized Cox modeling results in terms of estimated cumulative incidence of COVID-19 (from 7 to 92 days post-D57) across D57 marker levels. For each antibody marker, COVID-19 cumulative incidence/risk decreased as antibody marker level increased. Across the range of D57 spike IgG concentrations, estimated COVID-19 risk decreased from 0.023 (0.0072, 0.10) at low concentration spike IgG = 21 BAU/ml (2.5th percentile) to 0.0035 (0.0010, 0.011) at 1000 BAU/ml (96.5th percentile), a 6.6-fold change in risk level (Fig. 4a). For D57 nAb ID50, estimated COVID-19 risk decreased from 0.010 (0.0044, 0.038) at low nAb ID50 titer = 10 IU50/ml (8th percentile) to 0.0017 (0.0003, 0.0075) at 270 IU50/ml (97.5th percentile), a 5.9-fold change in risk level (Fig. 4b).

When vaccine recipients were divided into subgroups defined by their D57 antibody marker level above a specific threshold and the threshold was varied over the range of values, nonparametric regression showed that the cumulative incidence of COVID-19 (from 7 to 92 days post-D57) decreased monotonically as a function of each of the D57 markers (Fig. 4c, d). Risk decreased from 0.010 (0.004, 0.013) in all vaccine recipients to 0.00087 (0, 0.0025) for vaccine recipients with spike IgG concentration >500 BAU/ml (11.5-fold reduction), and to 0.0024 (0, 0.010) for vaccine recipients with nAb ID50 > 130 IU50/ml (4.2-fold reduction). Risk decreased more steeply over the span of positive antibody levels for IgG spike than for nAb ID50, and there is a sharp drop in risk when thresholding nAb ID50 above the detection limit, a feature not seen for IgG spike. Supplementary Fig. 6 provides corresponding tables of risk estimates.

### Vaccine efficacy increases with D57 spike IgG concentration and neutralization ID50 titer

**Fig. 5:**
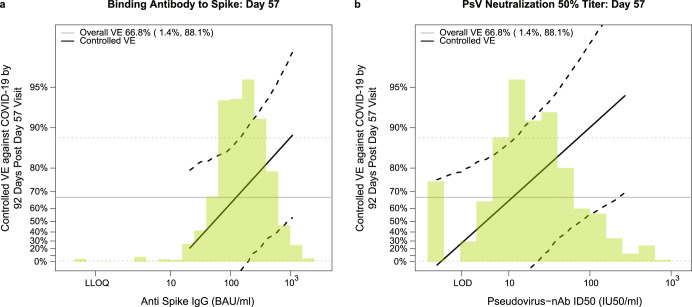
Vaccine efficacy by D57 antibody marker level in baseline SARS-CoV-2 negative vaccine recipients. Curves shown are for D57 (**a**) anti-spike IgG concentration or (**b**) pseudovirus (PsV)-nAb ID50 titer. The dotted black lines indicate bootstrap pointwise 95% confidence intervals. The green histogram is an estimate of the density of D57 antibody marker level and the horizontal gray line is the overall vaccine efficacy from 7 to 92 days post D35, with the dotted gray lines indicating the 95% confidence intervals. Analyses adjusted for age and baseline risk score. Curves are plotted over the antibody marker range from the 2.5th percentile to the 97.5th percentile: 21.3 to 1088 BAU/ml for anti-spike IgG and 1.31 to 270 IU50/ml for PsV-nAb ID50.

Figure 5 shows estimated vaccine efficacy against COVID-19 (from 7 to 92 days post-D57) across a range of levels of each D57 antibody marker [see the SAP (Section 11) and ref. ^28^. for the definition of vaccine efficacy]. Estimated vaccine efficacy increased with the level of each D57 marker. At D57 IgG concentration values of 21, 100, and 1000 BAU/ml (the 2.5th, 29th, and 96.5th percentiles), estimated vaccine efficacy against COVID-19 was 21.1% (95% CI: -361%, 79.3%), 62.9% (-55.0%, 85.7%), and 88.1% (52.0%, 97.0%), respectively. The analysis also showed that the D57 IgG concentration values that would be expected to achieve 50%, 70%, and 90% VE were 54.3 BAU/ml, 157 BAU/ml, and 1496 BAU/ml, respectively. At D57 nAb ID50 titers below the limit of detection (<2.612 IU50/ml), 10, 100, and 270 IU50/ml (the 8th, 27.5th, 89th, and 97.5th percentiles), these estimates were −5.8% (−651%, 75.6%), 64.9% (56.4%, 86.9%), 90.0% (55.8%, 97.6%) and 94.2% (69.4%, 99.1%). The D57 nAb ID50 titers that would be expected to achieve 50%, 70%, and 90% VE were 5.3 IU50/ml, 13.5 IU50/ml, and 99.9 IU50/ml, respectively.

A causal sensitivity analysis using the same methodology and implementation as that used in the correlates analyses of the COVE^23^, ENSEMBLE^24^, and PREVENT-19^25^ trials supported that vaccine efficacy increased with each D57 marker after accounting for potential unmeasured confounding of the effect of the D57 antibody marker on occurrence of COVID-19 (Supplementary Fig. 7).

### Comparing the antibody markers as correlates of risk and protection across phase 3 trials/vaccine platforms

We next compared the correlates of risk and the vaccine efficacy-by-antibody marker curves from the US/LatAm AZD1222 trial to those estimated from four other randomized, placebo-controlled COVID-19 vaccine efficacy trials: COVE (two doses of Moderna mRNA-1273 at D1 and D29)^29^, ENSEMBLE (one dose of Janssen Ad26.COV2.S at D1)^30^, COV002 in the United Kingdom (two doses of AstraZeneca AZD1222/ChAdOx1 nCoV-19 at D1 and D29)^2^, and PREVENT-19 (two doses of Novavax NVX-CoV2373 at D0 and D21)^31^. Supplementary Table 4 compares the primary efficacy endpoints of the five trials, with the major difference being the exclusion of mild COVID-19 from the ENSEMBLE primary endpoint. Antibody markers were measured 4 weeks post dose two, 4 weeks post dose one, 4 weeks post dose two, and 2 weeks post dose two, respectively. For ENSEMBLE we restricted comparison to data from the US study sites (ENSEMBLE-US) because the correlates analyses of PREVENT-19 and COVE were both restricted to the US data. For the US/LatAm AZD1222 trial, we included all of the data because 81% (82%) of vaccine cases for Day 29 (Day 57) correlates analyses were U.S. participants. The results at a given antibody marker level can be directly compared across the five trials due to conversion of both assay readouts to standardized international units^32,33^, as well as concordance testing of the Duke and Monogram neutralizing antibody assays^23,34^ (details in Methods).

**Fig. 6:**
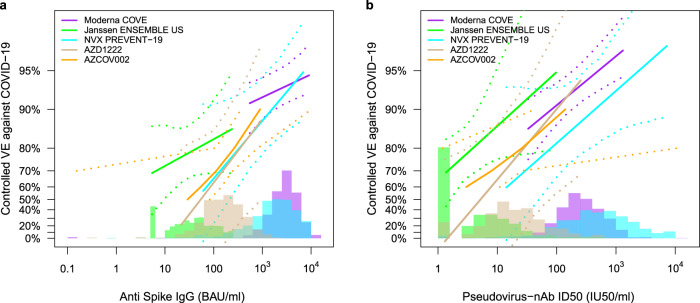
Vaccine efficacy (solid lines) in baseline SARS-CoV-2 negative participants by post-vaccination antibody marker level in five randomized, placebo-controlled COVID-19 vaccine efficacy trials. Vaccine efficacy was estimated using the marginalized Cox proportional hazards implementation of Gilbert et al.^28^. Vaccine efficacy (VE) estimates are shown by (**a**) anti-spike IgG concentration [D57 in COVE, D29 in ENSEMBLE-United States sites (ENSEMBLE-US), D35 in PREVENT-19, D57 in COV002] or (**b**) pseudovirus (PsV)-nAb ID50 titer (D57 in COVE, D29 in ENSEMBLE-US, D57 in AZD1222, D35 in PREVENT-19, D56 in COV002). The dashed lines indicate bootstrap point-wise 95% confidence intervals. The follow-up periods for the VE assessment were: COVE (doses D1, D29), 7 to 100 days post D57; ENSEMBLE-US (one dose, D1), 1 to 53 days post D29; PREVENT-19 (doses D0, D21), 7–59 days post D35; AZD1222 (doses D1, D29), 7 to 92 days post D57; COV002 (doses D1, D29; 7 to ~150 days post D57). The histograms are an estimate of antibody marker density in baseline SARS-CoV-2 negative vaccine recipients. Curves are plotted over the following antibody marker ranges: (**a**) COVE: 2.5th percentile to 97.5th percentile of marker, ENSEMBLE-US: 2.5th percentile to 97.5th percentile, PREVENT-19: 2.5th percentile to 97.5th percentile, COV002: 29 to 899 BAU/ml; (**b**) COVE: 10 IU50/ml to 97.5th percentile of marker, ENSEMBLE-US: 2.5th percentile to 97.5th percentile, PREVENT-19: 2.5th percentile to 97.5th percentile, AZD1222: 2.5th percentile to 97.5th percentile, COV002: 3 to 140 IU50/ml. Baseline covariates adjusted for were: COVE: baseline risk score, comorbidity status, and Community of color status; ENSEMBLE-US, baseline risk score; PREVENT-19: baseline risk score; AZD1222: age, baseline risk score; COV002: baseline risk score.

Table 3 compares the inverse correlates of risk results for spike IgG and for nAb ID50 across the four USG-supported trials. The estimated strength of the IgG spike correlate of risk is comparable in the PREVENT-19 and US/LatAm AZD1222 trials (HR per 10-fold increase = 0.36 and 0.32, respectively) and stronger than those in the COVE and ENSEMBLE-US trials (HR = 0.66 and 0.62, respectively). The estimated strength of the nAb ID50 correlate of risk is strongest in the US/LatAm AZD1222 trial (HR per 10-fold increase = 0.28) and comparable across the other three trials (HR 0.42, 0.38, 0.39 for COVE, ENSEMBLE-US, and PREVENT-19, respectively). Each antibody marker passed FWER-correction for being a significant correlate in 3 of the 4 trials. Estimated vaccine efficacy increased with increasing spike IgG concentration for all trials (Fig. 6a). For both spike IgG and nAb ID50, the vaccine efficacy curves are remarkably similar for the two ChAdOx1 nCoV-19 trials, providing clear replication of the correlates findings. The distribution of spike IgG is highest and similar for the NVX-CoV2373 and mRNA-1273 vaccines, intermediate for the AZD122 vaccine, and lowest for the Ad26.COV2.S vaccine. The vaccine efficacy curve is similar for the ChAdOx1 nCoV-19 and NVX-CoV2373 vaccines. The ChAdOx1 vaccine efficacy curves are to the right of the Ad26.COV2.S vaccine efficacy curve, suggesting that a lower IgG concentration may be required for the same level of vaccine efficacy for the Ad26.COV2.S vaccine.

**Table 3:** Comparison of correlate of risk results for spike IgG and PsV-nAb ID50 across four randomized, placebo-controlled COVID-19 vaccine efficacy trials (U.S. study sites).

Vaccine Platform^a^	Trial	Ab Marker 4 Wks Post-Vaccination	Follow-Up Post Vaccination	Estimated Hazard Ratio per 10-fold Increase in the Marker (95% CI)	*P*-Value	*Q*-Value	FWER-Adjusted *P*-Value
mRNA	COVE^23^	Spike IgG	126 days	0.66 (0.50, 0.88)	0.005	0.014	0.010
Ad26	ENSEMBLE-US^24^	Spike IgG	83 days	0.62 (0.28, 1.37)	0.24	0.35	0.36
Recombinant Protein	PREVENT-19^25^	Spike IgG	73 days	0.36 (0.20, 0.64)	<0.001	0.005	0.005
Ad (chimpanzee)	AZD1222	Spike IgG	92 days	0.32 (0.14, 0.76)	0.009	0.017	0.015
mRNA	COVE^23^	PsV-nAb ID50	126 days	0.42 (0.27, 0.65)	<0.001	0.002	0.003
Ad26	ENSEMBLE-US^24^	PsV-nAb ID50	83 days	0.38 (0.13, 1.12)	0.078	0.22	0.20
Recombinant Protein	PREVENT-19^25^	PsV-nAb ID50	73 days	0.39 (0.19, 0.82)	0.013	0.032	0.030
Ad (chimpanzee)	AZD1222	PsV-nAb ID50	92 days	0.28 (0.10, 0.77)	0.013	0.019	0.016

^a^COVE: Moderna mRNA-1273 spike vaccine; ENSEMBLE: Janssen Ad26 vector spike vaccine Ad26.CoV2.S; PREVENT-19: Novavax recombinant spike protein vaccine NVX-CoV2373; AZD1222: AstraZeneca ChAdOx1 nCoV-19 vaccine.

Estimated vaccine efficacy also increased with increasing nAb ID50 titer in each trial (Fig. 6b). The vaccine efficacy curve is similar for the ChAdOx1 nCoV-19 and mRNA-1273 vaccines, suggesting that the same neutralizing antibody titer is connected to approximately the same degree of efficacy of these vaccines.

### Spike IgG concentration and neutralization ID50 titer at D29 have no evidence as correlates of risk or protection

The same analyses described above were applied to the spike IgG and nAb ID50 markers measured at D29. Because D29 correlates analyses include cases between 7 days post D29 and up to 6 days post D57 that are not included in D57 correlates analyses (Supplementary Fig. 2), there are many more vaccine breakthrough cases: 69 compared to 33 for the D57 correlates analyses (Supplementary Figs. 1, 3). The results were completely flat: no evidence of correlates (Supplementary Tables 5, 6; Supplementary Figs. 8, 10–12). For example, the geometric mean D29 antibody levels were similar in vaccine non-cases vs. vaccine cases: spike IgG geometric mean ratio 0.97 (0.68, 1.4); nAb ID50 geometric mean ratio 0.99 (0.64, 1.5). Moreover, the hazard ratios of COVID-19 over 7–117 days post D29 per 10-fold increment in D29 antibody level were 1.01 (0.66, 1.55), *p* = 0.95 for spike IgG and 1.02 (0.67, 1.57), *p* = 0.91 for nAb ID50.

### Change in neutralization ID50 titer between D29 and D57 demonstrates evidence as correlates of risk or protection

Based on a reviewer’s suggestion, we used the same correlates of risk analyses as for the D57 markers to study the fold-rise in antibody markers before vs. after the second dose (i.e., log10 D57 markers – log10 D29 markers) as an immune correlate to explore antibody dynamics as a biomarker (Supplementary Table 8 and Supplementary Fig. 13). The analysis revealed a significant association between fold-rise and risk of COVID-19 for neutralization ID50 titer, with higher fold-rises associated with lower risk of COVID-19 [hazard ratio per SD increment in log10 fold-rise of 0.40 (0.26, 0.62), *p* < 0.001], but not for spike IgG [hazard ratio per SD of 0.62 (0.27, 1.44), *p* = 0.263].

## Discussion

The immune correlates analysis of baseline SARS-CoV-2 negative participants in the US/LatAm AZD1222 trial of the ChAdOx1 nCoV-19 COVID-19 vaccine vs. placebo showed that both anti-spike IgG concentration and pseudovirus neutralization ID50 titer measured 4 weeks post second dose (Day 57 study visit) were significant inverse correlates of risk of symptomatic COVID-19 occurrence over the subsequent 7–92 days. In addition, vaccine efficacy increased with higher D57 antibody marker levels, with estimated vaccine efficacy of 21% for vaccine recipients with low spike IgG value of 21 BAU/ml (2.5th percentile), increasing to 88% at 1000 BAU/ml (96.5th percentile). Moreover, estimated vaccine efficacy was 65% for vaccine recipients with low nAb ID50 value of 10 IU50/ml (8th percentile), increasing to 94% at 1000 IU50/ml (97.5th percentile). At undetectable nAb ID50 titer <2.612 IU50/ml, estimated vaccine efficacy was close to zero, −6%, but the confidence interval was wide due to the small percentage of vaccine recipients with undetectable neutralization titers at D57. A result with vaccine efficacy zero at undetectable titer is a favorable property of a correlate of protection because it implies a positive response is necessary for vaccine protection and supports that a large proportion of vaccine efficacy is mediated through the marker.

The two markers appeared to have similar strength as correlates of risk, for example with similar hazard ratios of COVID-19 for every SD increase in D57 marker, and similar widths of confidence intervals. Similarly, our ad-hoc analysis of fold rise in nAb ID50 from D29 to D57 indicated this marker also had a similar strength as a correlate of risk. The nonparametric threshold analysis (Fig. 4c, d) suggested that spike IgG may be a more discriminating correlate for differences in antibody levels in the medium to high range, whereas nAb ID50 seems to especially discriminate vaccine efficacy between vaccine recipients with undetectable vs. detectable D57 neutralization titer.

The perfect correlate of protection would fully mediate the overall vaccine efficacy, where mediation can be studied with causal inference methods that estimate the proportion of vaccine efficacy mediated through an immune marker. However, because ChAdOx1 nCoV-19 induced antibody responses by D57 in almost all vaccine recipients (positive spike IgG for 98.8% of vaccine recipients and detectable nAb ID50 titers for 92.0% of vaccine recipients), it was not possible to assess the proportion of vaccine efficacy mediated through either of these markers.

Interestingly, while both antibody markers were strongly supported to be correlates of protection when measured at D57, these markers did not correlate with protection when measured at D29, with no evidence whatsoever for being correlates. The statistical correlation of each antibody marker was low-moderate between the two time points (Spearman rank correlation 0.54 for spike IgG and 0.45 for nAb ID50), which makes the discordant result possible. If the markers had much lower inter-vaccine recipient variability at D29 than at D57, that could explain the discordant result, because marker dynamic range is a driver of power and precision for detecting correlates. Yet, the dynamic range of the markers was as high at D29 as at D57. Another potential explanation could be based on the inclusion of 36 early intercurrent COVID-19 endpoints in D29 correlates analyses that were not included in the D57 correlates analyses; however when the D29 correlates analyses were re-done excluding these early endpoints such that the same set of cases were included in each time point analysis, the correlates results were still completely flat (HR per 10-fold increase in bAb = 0.91 [0.50, 1.68]; HR per 10-fold increase nAb = 1.30 [0.78, 2.15]). In contrast, the COVE correlates study of the mRNA-1273 vaccine, a vaccine administered with the same Day 1, 29 dosing schedule, showed that the same two markers were slightly stronger correlates at D29 than at D57. These disparate results between the studies may be partly explained by the fact that the antibody levels are already well above the assay positivity/detection cut-offs (10.8424 BAU/ml/2.612 IU50/ml) at D29 in COVE (geometric means 318 BAU/ml and 13.0 IU50/ml^23^), whereas the levels are still near the positivity/detection cut-offs at D29 in the trial of the AZD1222 vaccine (geometric means 50 BAU/ml and 5.0 IU50/ml).

Further comparing across trials, the estimated ChAdOx1 nCoV-19 vaccine efficacy curves were similar in the AZD1222 and UK trials (Fig. 6)^26^, even though the latter trial had much greater variability in dosing interval; therefore dosing interval did not appear to modify the ChAdOx1 nCoV-19 correlate of protection. Moreover, the estimated ChAdOx1 nCoV-19 and mRNA-1273 vaccine efficacy curves were similar over the range of sufficiently overlapping neutralization titers (about 50 to 270 IU50/ml) where the curves can be compared, an encouraging result for potential cross-platform immunobridging and perhaps surprising given the substantial differences in vaccine regimens including a wild type vs. stabilized spike protein. Overall, the comparative results are consistent with the hypothesis that differences in overall vaccine efficacy across the four vaccine regimens may be explained by differences in the spike IgG and nAb ID50 induced by the vaccines. These results are also consistent with the results of immune correlates meta-analyses^35,36^.

The correlates analysis was done in the context of circulating SARS-CoV-2 strains that were all of the original Wuhan/Ancestral lineage or were early variants of concern or variants of interest that only had minor differences phylogenetically from Ancestral-lineage strains^37^. Based on Nextstrain.org^38^, we estimated the prevalence of circulating lineages during the periods of participant follow-up for correlates assessment (starting 7 days post D57) in the U.S. and Peru: U.S. more than 99% Ancestral lineage; and Peru 60% Lambda (C.37) variant, 21% Ancestral lineage and 6% Gamma (P.1) variant. No Global data sequences were found for Chile during the participant follow-up period. Additional research is needed to understand how the correlates of protection perform against SARS-CoV-2 strains that are more genetically and antigenically divergent from the Ancestral-lineage vaccine strain.

Strengths of the study include the randomized, double-blinded design and pre-specification of analyses that makes *p*-values and confidence intervals valid. Another strength is the harmonized design and analysis^21^ of the AZD1222, COVE, ENSEMBLE, and PREVENT-19 correlates studies, with harmonized trial protocols, restriction of the analysis to baseline SARS-CoV-2 negative participants, the use of a two-phase case-cohort antibody marker sampling design, application of the same reproducible and open source statistical methods, and the use of validated immunoassays with readouts for data analysis placed on the same WHO International Units scale. These harmonized elements enable comparability of results^21^.

We consider some additional limitations of this correlates study. First, other vaccine-induced immune responses of interest, such as spike-specific T-cell responses^39^, Fc effector antibody functions^40^, and memory B cells, were not assessed. These other potential mechanisms of protection may be particularly important in the context of hybrid immunity. The scope of this immune correlates analysis was limited to baseline SARS-CoV2 negative individuals and the results may not generalize to populations with high prevalence of previous infection with SARS-CoV-2 and hence with antibodies generated by both prior infection and vaccination. It was also not possible to study immune correlates for demographic subgroups of interest, nor in individuals previously infected with SARS-CoV-2 or in individuals who also received another SARS-CoV-2 vaccine. Other limitations include the relatively short follow-up (3 months post dose two), which precluded assessment of correlates for COVID-19 more distal from the primary vaccination series; the fact that 27% (12 of 45) of the vaccine breakthrough cases were missing data on antibodies at D57; the right-censoring of follow-up of 9.14% (95% CI: 8.4%, 9.91%) of vaccine recipients due to outside vaccination, and the fact that the case definition varied somewhat across the five vaccine trials, which limited the cross-study comparison. If the 12 cases missing antibody data had a different distribution of antibody levels than the 33 cases with antibody data after accounting for age and baseline risk score it would bias the results, and it was not possible to scrutinize this assumption. If outside vaccination or other types of loss to follow-up depended on participant factors associated with COVID-19 other than age and baseline risk score it could bias results. Moreover, due to the fact that none of the 33 vaccine breakthrough endpoints were severe, analysis was limited to a correlate against symptomatic disease, not severe disease. While the point estimates of vaccine efficacy against symptomatic disease varied across the four studied vaccines in the clinical trials^2,29–31^, large population effectiveness studies and meta-analyses suggest that all the vaccines are highly effective against severe disease/hospitalization and death against pre-Omicron viruses^41–44^.

Another important limitation is the limited genetic diversity of SARS-CoV-2 viruses amongst COVID-19 cases in our study, with continued effort needed to understand whether and how our results generalize to new variants of SARS-CoV-2. Progress in this direction stems from Cromer et al.^45^ and preceding papers from the Davenport group that developed a model to predict vaccine effectiveness for a given COVID-19 vaccine regimen, time period, and variant, based on the convalescent-sera standardized geometric mean titer of neutralizing antibodies to the variant during the time period, and have validated a high correlation of neutralization-CoP-model predicted vs. observed vaccine effectiveness, with some underestimation in prediction for the ChAdOx1 nCoV-19 vaccine (Supplementary Fig. 3 of Cromer et al.^45^). This model can be thought of as a ‘variant-invariant CoP model’ (term from Jerry Sadoff), which states that the relationship between vaccine protection against a given variant over a given time frame post-vaccination and the neutralizing antibody titer to that variant aggregated over that time frame, is the same across variants. The Cromer et al. neutralization-CoP model is based on the ‘vaccine-comparison’ or ‘population-level’ approach whereas the current manuscript uses the ‘individual-breakthrough’ approach; Khoury et al.^20^ showed close alignment in the two CoP modeling approaches (Fig. 2b for the ChAdOx1 nCoV-19 vaccine). Consequently, our Fig. 5b of vaccine efficacy vs. nAb ID50 titer can be interpreted as providing estimates of vaccine efficacy against a variant, based on changing the nAb ID50 value on the x-axis from data showing how the nAb ID50 titer of the vaccine regimen against the variant changes compared to that of the 2-dose ChAdOx1 nCoV-19 vaccine regimen against D614G, and with vaccine efficacy considered during the same time frame of follow-up post-vaccination as in the AZD1222 trial, and if necessary adjusted to account for any differences in antibody kinetics. For example, if participants in the AZD1222 trial were given a ChAdOx1 nCoV-19 booster dose, and post booster dose neutralizing antibody titer measured against BA.4/5 with a device available to standardize the readouts relative to the two-dose ChAdOx1 nCoV-19 response against D614G, then under the variant-invariant CoP model presumed to transport from 2 doses to 3 doses, we would expect the relationship in this figure to approximately represent the three-dose vaccine efficacy against BA.4/5 by titer against BA.4/5 after multiplying each x-axis value by 14.4. The factor 14.4 is derived based on Munro et al.^46^ and Liu et al.^47^ that studied ChAdOx1 nCoV-19 boosted recipients of the two-dose ChAdOx1 nCoV-19 primary series, with respective findings (1) an ~2.5-fold increase in nAb ID50 titer post booster compare to post dose 2; and (2) an ~36-fold decrease in titer against BA.4/5 compared to against the D614G strain (14.4 is 36 divided by 2.5).

Overall, this work provides an immune correlates analysis of a second phase 3 trial of the ChAdOx1 nCoV-19 vaccine, replicating the finding^26^ that both binding and pseudovirus neutralizing antibody titers are a correlate of protection for this vaccine, another step forward toward validating a surrogate endpoint for this vaccine.

## Methods

### Trial design, study cohort, COVID primary endpoints, and case/non-case definitions

Screening for enrollment for the US/LatAm AZD1222 trial (NCT04516746) began on August 28, 2020. A total of 32,451 participants met the eligibility criteria and were randomized (2:1 ratio) to receive two doses of either AZD1222 or placebo, one each on Days 1 and 29. Serum samples were taken on D1, D29, and D57 for antibody measurements in subset analyses. D29 and D57 antibody measurements were evaluated as correlates against adjudicated SARS-CoV-2 RT-PCR–positive symptomatic illness (COVID-19) endpoints.

The correlates analysis included COVID-19 endpoints up to the data cut-off date of March 5, 2021 (the same data cut-off date as that of the primary efficacy analysis). Correlates analyses were performed in baseline SARS-CoV-2 negative participants who received both planned vaccinations without any specified protocol deviations, and who were SARS-CoV-2 negative at the terminal vaccination visit (Supplementary Fig. 1). Within this correlates analysis cohort, intercurrent cases were COVID-19 endpoints in vaccine recipients starting 7 days post D29 through 6 days post D57, post Day 57 cases were COVID-19 endpoints in vaccine recipients starting 7 days post Day 57 through to the data cut-off (March 5, 2021), and non-cases/controls were vaccine recipients sampled into the immunogenicity subcohort with no evidence of SARS-CoV-2 infection (i.e., never tested RT-PCR positive) through to the data cut-off (March 5, 2021).

### Solid-phase electrochemiluminescence S-binding IgG immunoassay (ECLIA)

A validated solid-phase electrochemiluminescence S-binding IgG immunoassay was used to quantitate serum IgG binding antibodies against spike (homologous vaccine strain antigen, i.e. Wuhan-Hu-1)^23^. Assays were performed by Nexelis. Bound antibodies were detected using the Meso Scale Discovery (MSD) SULFO-TAG anti-human IgG antibody (mouse monoclonal, Meso Scale Diagnostics, LLC, Cat. No. D21ADF-3, 200x stock diluted 200-fold to prepare 1x solution). The MSD MESO Sector S 600 detection system was used to quantitate the amount of light emitted. The electrochemiluminescence unit response was reported as a result for each test sample, control sample and reference standard of each plate. The system software (MSD Discovery Workbench) is proprietary to MSD. Data analysis was performed with the Molecular Devices software, SoftMaxPro GxP, Version 6.5.1. Within an assay run, each human serum test sample was added to the precoated wells in duplicates in an 8-point dilution series. Readouts in arbitrary units/ml (AU/ml) were converted to bAb units/ml (BAU/ml) based on the World Health Organization 20/136 anti SARS-CoV-2 immunoglobulin International Standard^32^ as described^23^. Assay limits are provided in Supplementary Table 9. Antibody seroresponse was defined as IgG concentration above the positivity cut-off, 10.8424 BAU/ml. Values below the lower limit of quantitation (LLOQ = 1.35 BAU/ml) were set to LLOQ/2.

The same MSD assay was used in the PREVENT-19 trial^25^, and assay readouts were similarly converted to BAU/ml as above. Likewise, readouts (in Arbitrary Units/ml) of the MSD assay at VRC that was used in COVE^23^ and ENSEMBLE^24^ and the MSD assay at PPD that was used in COV002^26^ were transformed to the BAU/ml scale^32,33^. The fact that all spike IgG readouts are expressed in the same standardized units supports comparison of results at a given spike IgG concentration in Fig. 6.

### Pseudovirus neutralization assay

A validated assay^34^ using lentiviral particles pseudotyped with full-length SARS-CoV-2 spike (D614G strain that is almost identical to the vaccine strain) was used to measure neutralizing antibody titer as described^25^. Assays were performed by Monogram. As the limit of detection (LOD) was not formally defined, the LOD was set at the starting dilution level of the assay. Assay limits are provided in Supplementary Table 9. Neutralizing antibody response was defined by detectable ID50 (>LOD), with LOD = 2.612 IU50/ml. Values below the LOD were set to LOD/2. ID50 is reported in units (IU50/ml) calibrated to the 20/136 anti SARS-CoV-2 immunoglobulin International Standard.

Both the Duke pseudovirus neutralization assay (used in COVE) and the Monogram pseudovirus neutralization assay (used in this work) have undergone concordance testing^23,34^ and their readouts calibrated to the WHO International Standard^23,34^ to be expressed in standardized International Units (IU50/ml). For both the ENSEMBLE and PREVENT-19 trials, the same pseudovirus neutralization assay (D614G Monogram) was used to assay samples from each trial; the highly correlated ancestral D614 pseudovirus neutralization assay (Monogram) was used to assay samples from the COV002 trial^26^. All Monogram assay readouts were also calibrated to the WHO International Standard and are expressed in units of IU50/ml. The fact that all neutralizing antibody readouts are expressed in the same standardized units supports comparison of results at a given neutralizing antibody titer in Fig. 6.

### Inclusion and ethics

The US/LatAm AZD1222 trial protocol and all amendments were approved by the following local ethics committees and Institutional Review Boards: Chile: Universidad de Chile—Facultad de Medicina; Peru: El Comite Nacional Transitoria de Etica en Investigacion Para la Evaluacion y Supervision Etica de los Ensayos Clinicos de la Enfermedad; United States: WCGIRB, Oregon Health & Science University, Sutter Health Institutional Review Board, The University of Vermont Committees on Human Subjects, The Ohio State Biomedical Sciences Institutional Review Board, Columbia University.

All participants provided written informed consent prior to enrollment.

### Statistical methods

All data analyses were performed as pre-specified in the immune correlates SAP (Supplementary Material).

#### Case-cohort set included in the correlates analyses

A case-cohort^48^ sampling design was used to randomly sample participants for D1, D29, D57 antibody marker measurements. This random sample was stratified by the following baseline covariates: randomization arm, baseline SARS-CoV-2 status, and 6 baseline demographic covariate strata defined by all combinations of: minority vs. non-minority or unknown (where non-minority is defined as White non-Hispanic), age 18–64 vs. age ≥ 65, and enrollment at a US site vs. Chile or Peru site (see the SAP for details). Because the antibody markers were not measured in all participants due to this sub-sampling design and to happenstance missing data for some vaccine breakthrough cases, all of the statistical methods described below are corrected for biased sampling by weighting observations by the reciprocal of the empirical probability that a participant had marker data measured (see the SAP for details on weight estimation).

#### Covariate adjustment

All correlates analyses adjusted for age and a baseline risk score defined as the logit of predicted COVID-19 risk built from machine learning of data from baseline SARS-CoV-2 negative placebo recipients, where the predicted outcome was occurrence of SARS-CoV-2 RT-PCR–positive symptomatic illness starting 7 days after the D57 visit. Ensemble machine learning was used to build this risk score, using age, sex, ethnicity, race, BMI, and country as input variables. The baseline risk score had weak ability to predict COVID-19, with cross-validated area under the ROC curve (CV-AUC) 0.537 for the placebo arm and AUC 0.623 for the vaccine arm (Supplementary Fig. 14).

#### Correlates of risk in vaccine recipients

All correlates of risk and protection analyses were performed in baseline SARS-CoV-2 negative participants with no evidence of SARS-CoV-2 infection through 6 days post D57 visit and not right-censored by D57. Separately for each D57 and each D29 marker, the age and baseline risk score-adjusted hazard ratio of COVID-19 (across marker tertiles, per 10-fold increase in the quantitative marker, or per standard deviation-increment increase in the quantitative marker) was estimated using inverse probability sampling weighted Cox regression models with 95% CIs and Wald-based *p*-values. These Cox model fits were also used to estimate marker-conditional cumulative incidence of COVID-19 through 92 days post-D57 (or 117 days post-D29) in baseline negative vaccine recipients, with 95% CIs computed using the percentile bootstrap. The Cox models were fit using the survey package^48^ for the R language and environment for statistical computing^49^. Point and 95% CI estimates about marker-threshold-conditional cumulative incidence were computed by nonparametric targeted minimum loss-based regression^50^.

#### Correlates of protection: controlled vaccine efficacy

For each marker, vaccine efficacy by D57 or by D29 marker level was estimated by a causal inference approach using age and baseline risk score-marginalized Cox proportional hazards regression^28^. A sensitivity analysis of the robustness of results to potential unmeasured confounders of the impact of antibody markers on COVID-19 risk was also conducted. The analysis specified a certain amount of confounding that made it harder to infer a correlate of protection and estimated how much vaccine efficacy increases with quantitative D57 or D29 antibody marker despite the specified unmeasured confounder (see Section 12.1.2 in the SAP for further details).

#### Hypothesis testing

For hypothesis tests for D57 or D29 marker correlates of risk separately, Westfall-Young multiplicity adjustment^51^ was applied to obtain false-discovery rate adjusted *p*-values and family-wise error rate (FWER) adjusted *p*-values. Permutation-based multiple-testing adjustment was performed over the correlates of risk analyses of the markers expressed quantitatively or binned into tertiles. All *p*-values were two-sided.

#### Missing values of D1, D29 or D57 antibody markers

For each of the two immunoassays separately, for the set of participants with antibody data measured at D57, missing antibody values at D29 or D1 were imputed by predictive mean matching, using as predictors all available antibody data across the time points D1, D29, and D57. Then, for the set of participants with antibody data at D29, missing antibody values at D1 were imputed by predictive mean matching, using as predictors all available antibody data across the time points D1 and D29. The imputations were done with the *mice* R package, with default method “pmm”. Single imputation was done.

#### Calibration of neutralizing antibody assay readouts across laboratories

Calibration of ID50 nAb titers between the Duke University neutralization assay (COVE trial samples) and the Monogram PhenoSense neutralization assay (COV002 and ENSEMBLE trial samples) was performed using the WHO Anti-SARS CoV-2 Immunoglobulin International Standard (20/136) and Approach 1 of Huang et al.^34^ (with arithmetic mean as the calibration factor), as described in the supplementary material of Gilbert, Montefiori, McDermott et al.^23^

#### Software and data quality assurance

The analysis was implemented in R version 4.0.3^49^; code was verified using mock data.

### Reporting summary

Further information on research design is available in the Nature Research Reporting Summary linked to this article.

## Supplementary information

Supplementary Information

REPORTING SUMMARY

## Data Availability

Data underlying the findings described in this manuscript may be obtained in accordance with AstraZeneca’s data sharing policy described at https://astrazenecagrouptrials.pharmacm.com/ST/Submission/Disclosure. Data for studies directly listed on Vivli can be requested through Vivli at www.vivli.org. Data for studies not listed on Vivli could be requested through Vivli at https://vivli.org/members/enquiries-about-studies-not-listed-on-the-vivli-platform/. AstraZeneca Vivli member page is also available outlining further details: https://vivli.org/ourmember/astrazeneca/.

## Code availability

All analyses were done reproducibly based on publicly available R scripts hosted on the GitHub collaborative programming platform (https://github.com/CoVPN/correlates_reporting2).
