# Supplementary material for: Immune correlates analysis of a phase 3 trial of the AZD1222 (ChAdOx1 nCoV-19) vaccine
Source: NPJ Vaccines. 2023 Mar 11;8:36. doi: 10.1038/s41541-023-00630-0 (PMC10005913; doi:10.1038/s41541-023-00630-0)
Supplement: Supplementary file 2 — REPORTING SUMMARY [file 41541_2023_630_MOESM2_ESM.pdf]

nature portfolio

Corresponding author(s): Peter B. Gilbert

Last updated by author(s): Feb 8, 2023

Reporting Summary

Nature Portfolio wishes to improve the reproducibility of the work that we publish. This form provides structure for consistency and transparency in reporting. For further information on Nature Portfolio policies, see our Editorial Policies and the Editorial Policy Checklist.

Statistics

For all statistical analyses, confirm that the following items are present in the figure legend, table legend, main text, or Methods section.

n/a	Confirmed	
☐	☒	The exact sample size (*n*) for each experimental group/condition, given as a discrete number and unit of measurement
☐	☒	A statement on whether measurements were taken from distinct samples or whether the same sample was measured repeatedly
☐	☒	The statistical test(s) used AND whether they are one- or two-sided<br>*Only common tests should be described solely by name; describe more complex techniques in the Methods section.*
☐	☒	A description of all covariates tested
☐	☒	A description of any assumptions or corrections, such as tests of normality and adjustment for multiple comparisons
☐	☒	A full description of the statistical parameters including central tendency (e.g. means) or other basic estimates (e.g. regression coefficient) AND variation (e.g. standard deviation) or associated estimates of uncertainty (e.g. confidence intervals)
☐	☒	For null hypothesis testing, the test statistic (e.g. *F*, *t*, *r*) with confidence intervals, effect sizes, degrees of freedom and *P* value noted<br>*Give P values as exact values whenever suitable.*
☒	☐	For Bayesian analysis, information on the choice of priors and Markov chain Monte Carlo settings
☒	☐	For hierarchical and complex designs, identification of the appropriate level for tests and full reporting of outcomes
☐	☒	Estimates of effect sizes (e.g. Cohen's *d*, Pearson's *r*), indicating how they were calculated

Our web collection on statistics for biologists contains articles on many of the points above.

Software and code

Policy information about availability of computer code

Data collection	For the SARS CoV-2 Spike assay: The MSD MESO Sector S 600 detection system quantitates the amount of light emitted and reports the ECL unit response as a result for each test sample, control sample and reference standard of each plate. The system software (MSD Discovery Workbench) is proprietary to MSD: https://www.mesoscale.com/en/products_and_services/software.
Data analysis	For the SARS CoV-2 Spike assay: Data analysis was performed with the Molecular Devices software, SoftMaxPro GxP, Version 6.5.1.<br>For the correlates analyses: The analysis was implemented in R version 4.0.3; code was verified using mock data.<br>All analyses were done reproducibly based on publicly available R scripts hosted on the GitHub collaborative programming platform (https://github.com/CoVPN/correlates_reporting2).

For manuscripts utilizing custom algorithms or software that are central to the research but not yet described in published literature, software must be made available to editors and reviewers. We strongly encourage code deposition in a community repository (e.g. GitHub). See the Nature Portfolio guidelines for submitting code & software for further information.

Data

Policy information about availability of data

All manuscripts must include a data availability statement. This statement should provide the following information, where applicable:

- Accession codes, unique identifiers, or web links for publicly available datasets
- A description of any restrictions on data availability
- For clinical datasets or third party data, please ensure that the statement adheres to our policy

Data underlying the findings described in this manuscript may be obtained in accordance with AstraZeneca's data sharing policy described at https://astrazenecagrouptrials.pharmacm.com/ST/Submission/Disclosure. Data for studies directly listed on Vivli can be requested through Vivli at www.vivli.org. Data for studies not listed on Vivli could be requested through Vivli at https://vivli.org/members/enquiries-about-studies-not-listed-on-the-vivli-platform/. AstraZeneca Vivli member page is also available outlining further details: https://vivli.org/ourmember/astrazeneca/.

Human research participants

Policy information about studies involving human research participants and Sex and Gender in Research.

Reporting on sex and gender	Information on participant sex distribution is provided in Supplementary Table 2.<br>Table 3 in the Statistical Analysis Plan describes the baseline subgroups that are analyzed, including male and female, referring to sex assigned at birth. Within baseline negative vaccine recipients, it was prespecified to compare antibody levels between male vs. female. However, these results are not included in the present article.<br>Sex-based correlates analyses were not performed due to the low number of primary endpoints across both sexes (33 vaccine recipient breakthrough COVID cases), which would reduce the statistical power for such analyses to such an extent that the conclusions would likely not be meaningful.
Population characteristics	The demographic and clinical characteristics of the baseline SARS-CoV-2 negative participants selected into the immunogenicity subcohort cohort are provided in Supplementary Table 2.
Recruitment	Participants were recruited by site study teams. The study's inclusion and exclusion criteria are detailed on p. 9-11 of the Appendix of Falsey et al. Participants came from a wide range of racial and ethnic backgrounds and included people at increased risk of SARS-CoV-2 infection [defined as "Adults whose locations or circumstances put them at appreciable risk of exposure to SARS-CoV-2 and Covid-19, based on available risk assessment contemporaneous to enrollment (believed to be at risk/exposure)", see the Appendix of Falsey et al.].<br>The fact that the trial was a randomized trial, with careful allocation concealment, minimizes the potential for selection bias. Randomization was used to minimize bias in the assignment of participants to vaccine groups, to increase the likelihood that known and unknown participant attributes (eg, demographic and baseline characteristics) were evenly balanced across vaccine groups, and to enhance the validity of statistical comparisons across vaccine groups. From the Protocol, available with Falsey et al.: "Neither the participant nor any of the investigators or Sponsor staff who are involved in the treatment or clinical evaluation and monitoring of the participants will be aware of the study intervention received." For statistical analyses, "All personnel involved in the analyses of the study will remain blinded until the primary DBL and protocol deviations are identified." (also from the study protocol).
Ethics oversight	The US/LatAm AZD1222 trial protocol and all amendments were approved by the following local ethics committees and Institutional Review Boards: Chile: Universidad de Chile –Facultad de Medicina; Peru: El Comite Nacional Transitoria de Etica en Investigacion Para la Evaluacion y Supervision Etica de los Ensayos Clinicos de la Enfermedad; United States: WCGIRB, Oregon Health & Science University, Sutter Health Institutional Review Board, The University of Vermont Committees on Human Subjects, The Ohio State Biomedical Sciences Institutional Review Board, Columbia University.

Note that full information on the approval of the study protocol must also be provided in the manuscript.

Field-specific reporting

Please select the one below that is the best fit for your research. If you are not sure, read the appropriate sections before making your selection.

☒ Life sciences ☐ Behavioural & social sciences ☐ Ecological, evolutionary & environmental sciences

For a reference copy of the document with all sections, see nature.com/documents/nr-reporting-summary-flat.pdf

Life sciences study design

All studies must disclose on these points even when the disclosure is negative.

Sample size	In general, approximately 25 evaluable (with Day 0 and Day 35 Ab marker data available) vaccine recipient cases are desired to achieve good precision for correlate of risk analyses. For instance, the HVTN 505 trial serves as a precedent where 25 evaluable vaccine recipient cases provided enough data to reasonably characterize correlates of risk for a preventive candidate HIV vaccine (Janes et al., 2017; Fong et al.,

2018; Neidich et al., 2019; Gilbert et al., 2020). In addition, simulation studies show that correlates analyses at 20 endpoints have notably lower precision. Table 7 in the SAP provides the minimum number of vaccine endpoints for each correlates analysis type.

Data exclusions

As stated in section 6 of the SAP: "In two-phase sampling data analysis nomenclature, the “phase 1 ptids” are the per-protocol individuals excluding individuals with a COVID failure event or any other evidence of SARS-CoV-2 infection < 7 days post Day 57 visit. The “phase 2 ptids” are then the subset of these phase 1 ptids in the immunogenicity subcohort with Day 1 and Day 29 and Day 57 Ab marker data available. Thus, marker data for the COVID endpoint cases outside the subcohort will not be used in immunogenicity analyses; these cases are excluded from immunogenicity analyses. Similarly, for Day 29 marker correlates analyses the “phase 1 ptids” are the per-protocol individuals excluding individuals with a COVID failure event or any other evidence of SARS-CoV-2 infection < 7 days post Day 29. The “phase 2 ptids” are then the subset of these phase 1 ptids in the immunogenicity subcohort with Day 1 and Day 29 Ab marker data available. Thus again, marker data for the COVID endpoint cases outside the subcohort will not be used in immunogenicity analyses; these cases are excluded from immunogenicity analyses.

Also, as stated in section 9.3 of the SAP: "For baseline sampling stratum x [(vaccine, placebo) × (demographic strata)], the IPS weight w57.x assigned to a non-case participant in stratum x is defined by ˆ w57.x = 1/ˆπ57(x) = Nx/nx, where Nx is the number of stratum x vaccine recipient non-cases in the Per-Protocol Baseline Negative (PPBN) cohort and nx is the number of these participants that also have Day 1, 29, and 57 marker data available, where participants with any evidence of SARS-CoV-2 infection before 7 days post Day 57 visit are excluded from the counts Nx and nx. For non-case participant i in the immunogenicity subcohort, ˆ w57.i = 1/ˆπ57(Xi) denotes the weight ˆ w57.x for this individual’s sampling stratum. All Post Day 57 cases are assigned sampling weight N1/n1 where N1 is the total number of vaccine recipient cases in the PPBN cohort restricting to cases with event time starting 7 days post Day 57, and n1 is the number of these participants that also had the Day 1, 29, and 57 markers measured, and again participants with any evidence of SARS-CoV-2 infection < 7 days post Day 57 visit are excluded from the counts Nx and nx. In terms of two-phase sampling data analysis nomenclature, for the Day 57 marker analyses “phase 1 ptids” are defined as the entire PPBN cohort except excluding participants with any evidence of SARS-CoV-2 infection < 7 days post Day 57 visit. The “phase 2 ptids” are then the subset of these phase 1 ptids with Day 1, 29, and 57 Ab marker data available.

Also, as stated in section 16 of the SAP: "Because the lentivirus-based pseudovirus neutralization assay uses an HIV backbone, the presence of anti-retroviral drugs in serum will give a false positive neutralization signal."....Therefore... participants with any of the samples at Day 1, 29, 57 positive for antiretroviral use are excluded from analyses, for all analyses that include pseudovirus neutralization."

For the binding antibody assay: Any plates and samples that did not meet the following acceptance criteria were excluded and repeated:
• Plate calibrator curve fit $R2 \geq 0.98$
• Calibrator replicate signal CV (coefficient of variation) and back-calculated concentration ≤ 20% for standards within LSL (lower sensitivity level) and USL (upper sensitivity level) range
• Calibrator replicate signal CV (coefficient of variation) and back-calculated concentration ≤ 25% for LSL and USL standards.
• Plate controls signal CV (coefficient of variation) ≤ 20%.
• Recoveries of plate controls within +/-30% of the nominal values for MSD-high, MSD-mid, and MSD-low. Recoveries of plate controls within +/-30% of the nominal values for Sercare-high, Seracare-Mid, and In-house prepared serum control. Recoveries of plate controls within +/-25% of the nominal values for Seracare-Negative.
• Sample replicate CVs ≤ 20%.

Replication

All of the immune correlates analyses are implemented in automated and reproducible press-button fashion. The analyses code are hosted in a github repo that is open to the public (https://github.com/CoVPN/correlates_reporting2).

For the binding antibody assay: Reproducibility was ensured by running 8 controls in all plates assayed including: MSD-high, MSD-mid, MSD-low, Sercare-high, Seracare-Mid, Seracare-Negative, and In-house prepared serum control sample.

Randomization

As stated in the study protocol (section 6.3.1), available with Falsey et al. NEJM, participants were "centrally assigned to randomized study intervention using an IRT. Before the study is initiated, user guides, the log in information, and directions for the IRT will be provided to each study site. Randomization will be stratified by age (≥ 18 to < 65 years, and ≥ 65 years), with at least 25% of participants to be enrolled in the older age stratum."

Blinding

As stated in the study protocol (section 6.3.2), available with Falsey et al. NEJM, "Neither the participant nor any of the investigators or Sponsor staff who are involved in the treatment or clinical evaluation and monitoring of the participants will be aware of the study intervention received. Since AZD1222 and placebo are visually distinct prior to dose preparation (due to differences in container closure), IMP will be handled by an unblinded pharmacist (or designee in accordance with local and institutional regulations) at the study site. Once drawn into syringes for administration, AZD1222 and placebo are not visually distinct from each other." Moreover, the treatment arm assignment was blinded to the labs running the assays for the correlates analyses.

Reporting for specific materials, systems and methods

We require information from authors about some types of materials, experimental systems and methods used in many studies. Here, indicate whether each material, system or method listed is relevant to your study. If you are not sure if a list item applies to your research, read the appropriate section before selecting a response.

Materials & experimental systems

n/a	Involved in the study
☐	☒ Antibodies
☐	☒ Eukaryotic cell lines
☒	☐ Palaeontology and archaeology
☒	☐ Animals and other organisms
☐	☒ Clinical data
☒	☐ Dual use research of concern

Methods

n/a	Involved in the study
☒	☐ ChIP-seq
☒	☐ Flow cytometry
☒	☐ MRI-based neuroimaging

Antibodies

Antibodies used: MSD SULFO-TAG anti-human IgG antibody (Meso Scale Diagnostics, LLC, Cat. No. D21ADF-3). Diluted 200-fold to prepare 1x solution from stock.

Validation: Certificates of analysis and technical notes are available at https://www.mesoscale.com/en/products/msd-gold-sulfo-tag-nhs-ester-r91ao/

Eukaryotic cell lines

Policy information about cell lines and Sex and Gender in Research

Cell line source(s): HEK-293 T; source: Master Cell Bank established by Monogram Biosciences circa 1996

Authentication: No formal authentication. Cell line in continuous use since establishment of Master Cell Bank.

Mycoplasma contamination: The HEK-293T cell line tested negative for mycoplasma contamination. Mycoplasma testing is routinely performed per MGRM SOP.

Commonly misidentified lines (See ICLAC register): None.

Clinical data

Policy information about clinical studies
All manuscripts should comply with the ICMJE guidelines for publication of clinical research and a completed CONSORT checklist must be included with all submissions.

Clinical trial registration: NCT04516746

Study protocol: The full trial protocol of Falsey et al. can be found with the full text of the primary article at NEJM.org: https://www.nejm.org/doi/suppl/10.1056/NEJMoa2105290/suppl_file/nejmoa2105290_protocol.pdf

Data collection: Clinical data collection is described in the primary publication, Falsey et al. NEJM: https://www.nejm.org/doi/full/10.1056/NEJMoa2105290at. As stated in Falsey et al., the study was conducted at 88 sites in the United States, Chile, and Peru. Participants were screened and randomized between August 28, 2020, and January 15, 2021 and the cutoff date for the primary analysis was March 5, 2021. The 88 clinical sites are listed below:
D8110C00001 CHL Quillota Centro Respiratorio Integral
D8110C00001 CHL Santiago Facultad de Medicina - Universidad de Chile
D8110C00001 CHL Santiago Hospital Luis Calvo Mackenna
D8110C00001 PER Cercardo de Lima Clinica Internacional - Lima
D8110C00001 PER La Perla Centro de Invetigaciones Medicas
D8110C00001 PER San Isidro Clinica Ricardo Palma
D8110C00001 USA Albuquerque MedPharmics Â– Albuquerque
D8110C00001 USA Ankeny The Iowa Clinic, PC
D8110C00001 USA Ann Arbor University of Michigan
D8110C00001 USA Atlantis JEM Research Institute
D8110C00001 USA Austin Tekton Research, Inc
D8110C00001 USA Baltimore Johns Hopkins Bloomberg School of Public Health
D8110C00001 USA Baltimore Pharmaron/SNBL Clinical Pharmacology Center
D8110C00001 USA Berkeley East Bay AIDS Center
D8110C00001 USA Berlin Hassman Research Institute
D8110C00001 USA Bethesda Walter Reed National Military Medical Center
D8110C00001 USA Bloomington HealthPartners Institute
D8110C00001 USA Boston Fenway Community Health
D8110C00001 USA Boston Tufts Medical Center, Inc. - PARENT
D8110C00001 USA Bronx Montefiore Medical Center
D8110C00001 USA Brooklyn NYU Langone Brooklyn
D8110C00001 USA Burlington The University of Vermont Medical Center
D8110C00001 USA Butte Mercury Street Medical Group

D8110C00001 USA Charleston Clinical Trials of South Carolina
D8110C00001 USA Charleston Medical University of South Carolina (MUSC)
D8110C00001 USA Chicago AYAR at CORE CRS
D8110C00001 USA Chicago Rush University Medical Center
D8110C00001 USA Cincinnati Cincinnati Children's Hospital Medical Center
D8110C00001 USA Columbus Ohio State University
D8110C00001 USA Coral Gables AMR Coral Gables/Miami, Formerly Clinical Research of South Florida, an AMR company
D8110C00001 USA Dallas Trinity Health and Wellness Center/AIDS Arms, Inc.
D8110C00001 USA Danbury Western Connecticut Health Network
D8110C00001 USA Denver University of Colorado at Denver and the Health Sciences
D8110C00001 USA Durham Duke University Health System
D8110C00001 USA El Centro ECRMC Women's Health Center
D8110C00001 USA Fairway University of Kansas Medical Center
D8110C00001 USA Fort Belvoir Fort Belvoir Community Hospital
D8110C00001 USA Gulfport MedPharmics LLC
D8110C00001 USA Honolulu East-West Medical Research Institute
D8110C00001 USA Houston University of Texas-Houston Hariss County Psychiatric Center
D8110C00001 USA Indianapolis Indiana University
D8110C00001 USA Kansas City University of Kansas Hospital
D8110C00001 USA Knoxville AMR Knoxville, Formerly New Orleans Center for Clinical Research - Knoxville, an AMR company
D8110C00001 USA Lackland AFB Joint Base San Antonio
D8110C00001 USA Lake Charles Centex Studies Inc.
D8110C00001 USA Lexington AMR Lexington, Formerly Central Kentucky Research Associates, an AMR company
D8110C00001 USA Little Rock Applied Research Center of Arkansas
D8110C00001 USA Los Angeles UCLA
D8110C00001 USA Los Angeles University of Southern California Medical Center
D8110C00001 USA Madison University of Wisconsin
D8110C00001 USA McAllen Centex Studies, Inc.
D8110C00001 USA Meridian Advanced Clinical Research
D8110C00001 USA Miami Lakes Case Clinical Research Site
D8110C00001 USA Mineola NYU Winthrop Hospital
D8110C00001 USA Monroe Clinical Trials of America LA, LLC
D8110C00001 USA Nashville Clinical Research Associates, Inc.
D8110C00001 USA New York Bellevue Hospital, Manhattan (NYU affiliate)
D8110C00001 USA New York Bronx Prevention Research Center CRS
D8110C00001 USA New York Columbia P&S CRS
D8110C00001 USA New York NYU Langone Health Center
D8110C00001 USA New York VA NY Harbor Healthcare System
D8110C00001 USA Oklahoma City Tekton Research
D8110C00001 USA Orlando Orlando Immunology Center
D8110C00001 USA Phoenix Hope Research Institute
D8110C00001 USA Pittsburgh University of Pittsburgh
D8110C00001 USA Portland Oregon Health & Science University
D8110C00001 USA Portsmouth ActivMed Practices & Research, Inc. - PORTSMOUTH
D8110C00001 USA Richmond Clinical Research Partners, LLC
D8110C00001 USA Rochester Rochester General Hospital
D8110C00001 USA Rochester University of Rochester Medical Center
D8110C00001 USA Rochester University of Rochester Medical Center
D8110C00001 USA Royal Oak Beaumont Health System
D8110C00001 USA San Antonio Brooke Army Medical Center PHARM
D8110C00001 USA San Diego Naval Medical Center San Diego
D8110C00001 USA San Diego UCSD Antiviral Research Center (AVRC)
D8110C00001 USA San Francisco Bridge HIV CRS
D8110C00001 USA San Francisco Zuckerberg San Francisco General Hospital
D8110C00001 USA Scottsdale Cognitive Clinical Trials, LLC
D8110C00001 USA Seattle Seattle Vaccine and Prevention CRS
D8110C00001 USA South Charleston West Virginia Research Institute
D8110C00001 USA Spartanburg Spartanburg Medical Research
D8110C00001 USA The Woodlands Javara Inc
D8110C00001 USA Torrance Lundquist Institute for Biomedical Innovation at Harbor-UCLA Medical Center
D8110C00001 USA Valhalla New York Blood Center
D8110C00001 USA Warwick Omega Medical Research
D8110C00001 USA West Jordan Advanced Clinical Research
D8110C00001 USA Wichita AMR East Wichita, Formerly Heartland Associates East Wichita, an AMR company
D8110C00001 USA Wichita KU Wichita Center for clinical research

Outcomes

The primary, secondary, and exploratory objectives and endpoints of the trial are all described in the Supplementary Appendix of the primary publication, Falsey et al. NEJM. The specific link to the Appendix is: https://www.nejm.org/doi/suppl/10.1056/NEJMoa2105290/suppl_file/nejmoa2105290_appendix.pdf.
The following text is taken from the Appendix:
For the primary endpoint, "symptomatic was defined as 1 or more of the following criteria: pneumonia diagnosed by chest X-ray or computed tomography scan; oxygen saturation 94% or less on room air or requiring new initiation or escalation in supplemental oxygen; or new or worsening dyspnea/shortness of breath; or 2 or more of the following symptoms/signs: fever over 100°F or feverishness; new or worsening cough; myalgia/muscle pain; fatigue that interferes with activities of daily living; vomiting and/or diarrhea (one finding counted toward endpoint definition); or anosmia and/or ageusia (one finding counted toward endpoint

definition). Participants who experienced one or more of the COVID-19 qualifying symptoms listed in the Appendix of Falsey et al. were instructed to contact the trial team."
"If a participant presented with Covid-19 qualifying symptom(s) on Days 1–3 postvaccination, the nasal pharyngeal (NP) swab that was collected on Day 1 was sent for local SARS-CoV-2 RT-PCR testing. If positive, the participant was instructed to initiate illness visits. If negative, the participant continued with scheduled assessments. After Day 3 postvaccination, a participant with Covid-19 qualifying symptoms was instructed to attend illness visit 1 where two NP swabs were collected, one for local RT-PCR testing and one for central testing. The local test was used for patient management and the central test was used to determine SARS-CoV-2 RT-PCR status. If the local RT-PCR was negative, the participant was directed to stop illness visits and resume regular follow-up visits. If positive, the participant continued with illness visits and was instructed in home collection requirements, including use of a digital health device, saliva samples, and e-Diary recordings. NP swabs for central lab RT-PCR were also collected at illness visits on Days 14, 21, and 28. In the event that the central lab PCR was not collected or was not available (i.e., lost in shipping, spoiled, etc.) then the local lab PCR result was used for endpoint determination. If the local and central PCR test results were discordant, such that the local was positive and the central was negative, the adjudication committee could consult the saliva RT-PCR result in determining whether the participant was PCR-positive. Serum samples were collected from participants at Days 1, 29, 57, 90, 180, 360, and 730 for SARS-CoV-2 antibody testing to monitor participants for interim acquisition of asymptomatic infection. Authorized laboratories assessed serologic responses to AZD1222 by the rate of participants seroconverting from negative to positive as defined by a validated immunoassay directed at the SARS-CoV-2 spike antigen. Additional serum samples were collected in the substudy at Days 15 and 43 for immunogenicity testing. Saliva was collected during illness visits and at home to quantify duration of viral shedding on Days 1, 3, 5, 8, 11, 14, 21, and 28 of the illness."
